# Family Factors Associated with Selected Childhood Nutrition Aspects in Central Poland

**DOI:** 10.3390/ijerph16040541

**Published:** 2019-02-13

**Authors:** Katarzyna Zadka, Ewelina Pałkowska-Goździk, Danuta Rosołowska-Huszcz

**Affiliations:** Department of Dietetics, Faculty of Human Nutrition and Consumer Sciences, Warsaw University of Life Sciences WULS-SGGW, 159c Nowoursynowska Str., 02-776 Warsaw, Poland; ewelina_palkowska_gozdzik@sggw.pl (E.P.-G.); danuta_rosolowska_huszcz@sggw.pl (D.R.-H.)

**Keywords:** diet-related diseases, family factors, breastfeeding, snack consumption, fast food consumption, fried food consumption, dental caries occurrence

## Abstract

Childhood diet has a significant influence on diet-related diseases in adulthood, so an understanding of environmental influences on nutrition, is important. The aim of this cross-sectional study was to indicate family factors associated with some aspects of children’s nutrition in Central Poland. A questionnaire was used to investigate 892 mothers’ approach to breastfeeding, frequency of eating with children at fast food restaurants, and serving them snacks, sugary drinks, and fried food. Prevalence of dental caries among children, based on the mothers’ self-assessment, was also assessed. Majority of the mothers breastfed for a period not longer than six months. There was a positive association between breastfeeding duration and mothers’ education level and the number of children in a family. Sweets were used as a reward, more often among younger children and in families with higher number of children. The frequency of consumption of sweet beverages rose with the child’s age and decreased with mother’s education level and family income. It was also more frequent in rural areas. Most children received snacks and fried food at least once a week. There was a negative association between eating with parents at fast food restaurants and, both, the number of children in the family and living in a village. Fast food consumption rose with the mother’s education level and family income. Prevalence of dental caries according to mothers’ declarations was much lower than in national studies but was associated with frequent consumption of snacks and sweet beverages in the examined population. Extensive activities to reduce the occurrence of dental caries at the national level and education concerning the role of a family environment in providing a proper childhood nutrition, with a special emphasis on breastfeeding benefits, seems necessary for Polish parents. Designing community-wide education campaigns referencing population-based programs and other health and disease prevention activities, need to be promoted.

## 1. Introduction

Chronic non-communicable diseases (NCDs), including cardiovascular diseases, cancers, chronic respiratory diseases, and diabetes, are major causes of mortality in the world. A growing body of evidence suggests that NCDs have a complex etiology and it is well-documented that chronic diseases in adulthood have origins in an early life [1]. Therefore, an understanding of children’s eating habits and behaviors is important for improving childhood health and its influence on health in adulthood. It is known that a child’s eating behavior is strongly influenced by the family environment. Parents may foster the development of healthy eating habits among children or may promote obesity and aspects of disorderly eating [2]. Mothers are of particular interest because they commonly spend significantly more time in direct interactions with the children, across several familial situations (including mealtimes), than does the fathers [3]. Mothers have a crucial role from the beginning, as they decide what they eat during pregnancy and the breastfeeding duration, which has short-term and long-term beneficial health effects at individual and population levels. Breastfeeding might protect against cardiovascular diseases [4], obesity [5,6], hypertension [7], high blood cholesterol [8], diabetes type 2 [9,10], and dental caries [11] in later life. In the later years of children’s life, their nutrition patterns depend highly on the parents’ eating habits. Important environmental factors associated with the future occurrence of NCDs are—improper eating habits, type of food available at home, level of consumption of fast foods, sweets and sweetened soft drinks, and many others [12]. It includes socio-demographic factors, parental activity, parental eating style, and parents style of child-feeding [13]. According to a study conducted on a group of 560 pupils, aged 8–13 years, children whose parents regularly consumed sweet beverages had a three-times greater propensity to consume these beverages, compared to peers whose parents did not regularly drink these beverages [14]. This high consumption was associated with a risk of obesity, due to the high free sugar content. It likely increases the risk of type-2 diabetes, as well [15]. Other research has showed that the availability of sweets in a child’s home and parental inappropriate modeling of eating were associated with an increased risk for consumption of these products by children. In addition, parental monitoring of the child’s eating was associated with a reduced risk for sweets intake, and a lower BMI [16]. Diets high in sugar have also been associated with dental caries [17]. Poland has one of the highest prevalence of dental caries among children in Europe. The oral hygiene condition of Polish children is unsatisfactory and worsens with age. According to epidemiological studies of the Polish Ministry of Health, cavities were present in 76.8% of children aged 5, and 89.4% of children of age 12 years and below. Unfortunately, an awareness concerning this issue among Polish parents is rather low [18]. Obesity among children and risk of future NCDs occurrence are assumed to also be the results of an extensive fat intake [19]. High consumption of saturated fats might influence some of the major risk factors for coronary heart disease, impair vasoactivity, and the endothelial function [20]. Fried food consumption has been reported to be positively associated with obesity and, thus, with related chronic diseases [21,22]. In many regions of the world, frying is a common and traditional cooking method, which is also used in numerous restaurants, especially the fast food ones [23]. Therefore, obesity and, thus, NCDs might also be connected with frequent consumption of food in fast food places and other restaurants. It has been established that children who ate at fast food restaurants, two or more times a week, were more likely to have an increased BMI, compared to those who ate fast food once a week or less often [24].

The aim of this cross-sectional study was to indicate family factors associated with some aspects of children’s nutrition in Central Poland. We investigated mothers’ approach to breastfeeding, frequency of eating with children at fast food restaurants, and serving them snacks, sugary drinks, and fried food. We also studied if this was related to the number of children in the family, place of residence, monthly net income per family, and the level of education of the mother. We also assessed prevalence of dental caries among children, based on the mothers’ self-assessment.

## 2. Materials and Methods 

Selection of the sample and methodology of the survey, which was carried out in Poland, between September 2016 and March 2017, among 892 mothers of children aged 7–14 years, has been described elsewhere [25]. Below, only questions which concerned the scope of this publication are depicted.

In a question concerning breastfeeding we asked for duration of breastfeeding, including any type of such feeding defined by the World Health Organization [26]. Mothers were also asked in a closed question if they offered children sweets, like chocolate blocks, chocolate bars, wafers, ice-creams, lollipops, and candies, as a reward. Further questions concerned eating with children at fast food restaurants and serving them sweet beverages, sweet and salty snacks, and fried food. Apart from declarations on the frequency of serving of sweet beverages and snacks, mothers were asked to indicate children’s favorite products from these categories. They also declared if a child had dental caries, based on their self-assessment.

For statistical analyses, the PAWS Statistics 18 software (SPSS Inc., Hong Kong) was used. The Goodman and Kruskal’s gamma (G) was run to determine the association between the analyzed parameters. Statistical significance of differences was estimated at *p* ≤ 0.05.

The study protocol was approved by the Ethics Committee of the Faculty of Human Nutrition and Consumer Sciences of the Warsaw University of Life Sciences (SGGW-WULS) in Warsaw, Poland (No. 11/2017).

## 3. Results

### 3.1. Characteristics of the Examined Mothers and Their Children

Characteristics of the examined mothers and their children, including prevalence of inadequate body mass among mothers and children, has been published elsewhere [25].

### 3.2. Breastfeeding 

**Figure 1:**
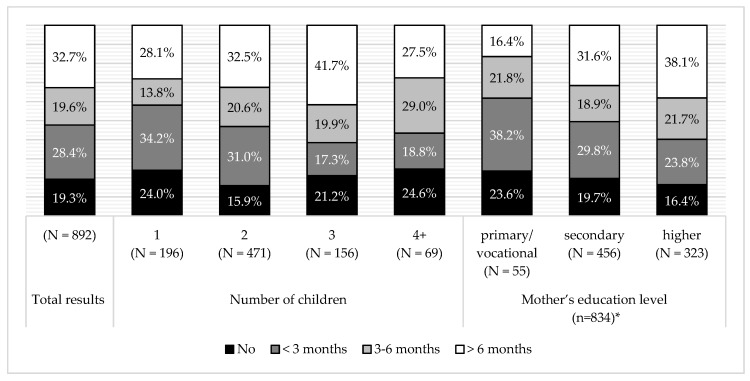
Breastfeeding in the total surveyed population and sub-groups, according to number of children and mother’s education level. * 58 mothers did not declare education level.

In the examined group, majority of the children were breastfed for a period not longer than six months. There was a positive association between breastfeeding duration and mothers’ education level (G = 0.159, *p* < 0.005). The more educated the mother was, the longer did she practice breastfeeding. Positive association was also observed between breastfeeding duration and the number of children in a family (G = 0.102, *p* < 0.05). Those mothers who had two or three children, were more likely to breastfeed for a longer duration. However, mothers who had four or more children did not breastfeed for as long as mothers of two or three children, but were still longer than mothers of only one child (Figure 1). There was no association between breastfeeding duration and child’s BMI or the prevalence of dental caries.

### 3.3. Using Sweets as a Reward and as a Check for the Prevalence of Dental Caries 

**Figure 2:**
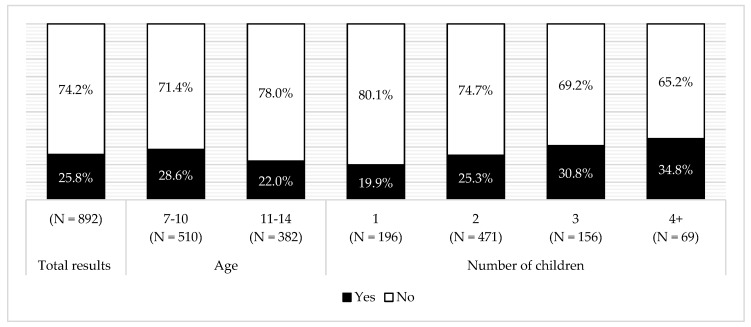
Using sweets as a reward in the total surveyed population and sub-groups, according to age and number of children.

Majority of examined mothers did not use sweets as a reward. There was a negative association between using sweets as a reward and child’s age (G = −0.175, *p* < 0.05). A positive association was observed between using sweets as a reward and the number of children (G = 0.182, *p* < 0.005) (Figure 2). It was also observed that children who more frequently received sweets as a reward were more likely to have dental caries (G = 0.213, *p* < 0.05). No association was observed between using sweets as a reward and the child’s BMI.

**Figure 3:**
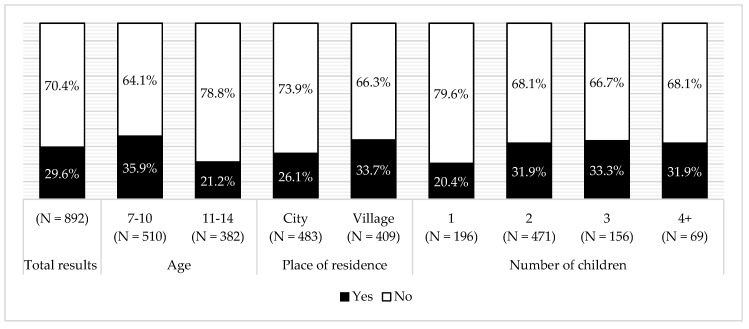
Dental caries prevalence in the total surveyed population and sub-groups, according to age, place of residence, and number of children.

The mothers themselves assessed their children’s deciduous and adult teeth. According to mother’s declarations, majority of the children had no caries issues. More mothers of younger children declared problems with caries presence. There was a strong, negative association between age and caries that were self-reported by mothers (G = −0.351, *p* < 0.0005); however, it could be affected by adult teeth eruption time. Additionally, living in a village was connected with more frequent declaration that a child had problems with teeth (G = 0.181, *p* < 0.05). Prevalence of caries also rose with the number of children in the family (G = 0.162, *p* < 0.05) (Figure 3).

### 3.4. Sweets Beverage and Snack Consumption

**Figure 4:**
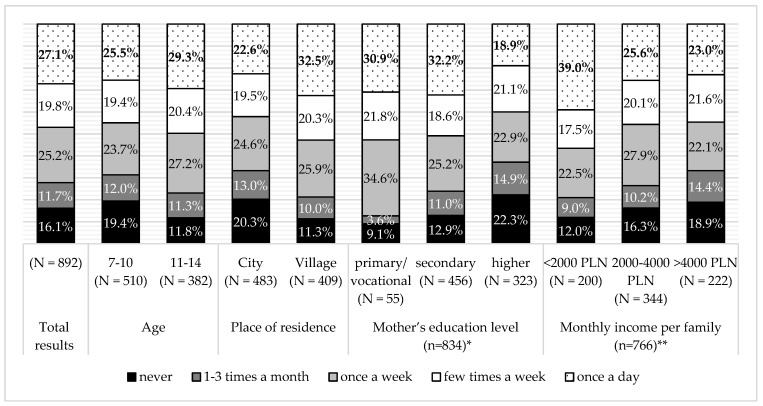
Sweet beverage consumption in the total surveyed population and sub-groups, according to age, place of residence, mother’s education level, and monthly income per family. * 58 mothers did not declare education level; ** 126 mothers did not declare income per family.

The frequency of sweet beverage consumption was very high. Consumption rose with age (G = 0.117, *p* < 0.05) and decreased with the mother’s education level (G = −0.220, *p* < 0.0005) and the net monthly family income (G = −0.150, *p* < 0.005). Sweet beverages were also much more often consumed by rural than urban children (G = 0.210, *p* < 0.0005) (Figure 4). Prevalence of dental caries rose with consumption of sweet beverages (G = 0.228, *p* < 0.0005). No association was observed between sweet beverage consumption and the child’s BMI. The most frequently indicated groups of sweet beverages in children’s diet were—flavored water (65%), cola type beverages (25%), and orangeade (19%). Flavored water was included because in Poland such beverages are a source of monosaccharides and disaccharides.

More than 80% of children received snacks at least once a week. A total of 28.9% of mothers served snacks once a day, 30.9% served snacks a few times a week, and 30.7% served only once a week. Only 7.3% of children received them 1–3 times a month and 2.1% never received any snacks. We did not observe any relation between serving snacks and BMI and any examined environmental factors. However, a positive association was observed between snack consumption and dental caries (G = 0.259, *p* < 0.0005). The most popular snacks among the examined families were chocolate-based sweets (bars, blocks) (67%), candies and lollipops (37%), and chips and French fries (26%). Snacks were served mainly in the afternoon.

### 3.5. Fried Food and Fast Food Consumption

**Figure 5:**
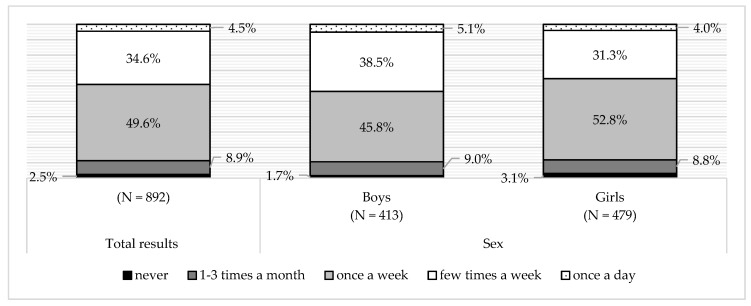
Fried food consumption in the total surveyed population and the sub-groups, according to sex.

Most children from the examined group consumed fried food, at least once a week. There was a negative association between sex (being a girl) and eating fried food (G = −0.133, *p* < 0.05) (Figure 5). No association was observed between fried food consumption and the child’s BMI.

**Figure 6:**
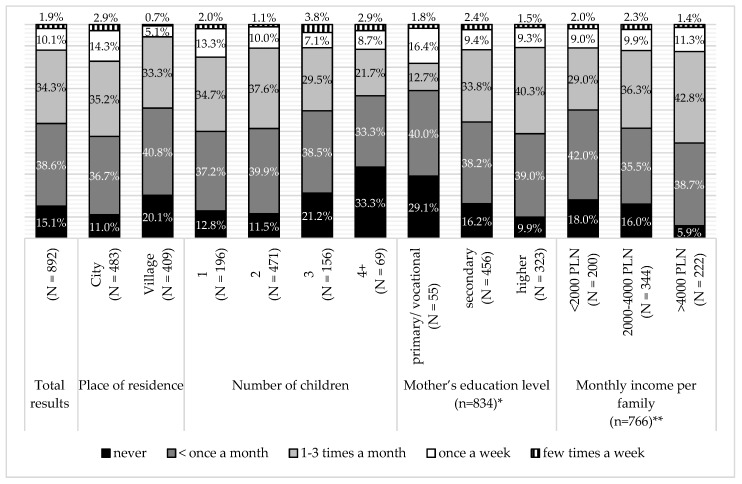
Consumption at fast food restaurants in the total surveyed population and the sub-groups, according to place of residence, number of children, mother’s education level, and monthly income per family. * 58 mothers did not declare education level; ** 126 mothers did not declare income level per family.

According to the mothers’ declaration, the majority of children visited fast food restaurants with one of the parents, less than once a month or few times a month. There was a negative association between frequency of visiting fast food restaurants and the number of children (G = −0.147, *p* < 0.005). Children from rural areas ate at such places, two times less often; there was a negative association between living in a village and fast food consumption (G = −0.282, *p* < 0.0005). On the other hand, frequency of eating at fast food restaurants with children rose with the mother’s education level (G = 0.132, *p* < 0.05) and net monthly family income (G = 0.163, *p* < 0.0005) (Figure 6). There was no association between visiting fast food restaurants with parents and the child’s BMI.

## 4. Discussion

Unfavorable aspects of nutrition-related behaviors were frequent among the examined population, however, their prevalence differed. Almost half of the mothers did not breastfeed at all or for no longer than three months, while sweets were used as a reward by only about one-quarter of them. At the same time, most of children received sweet beverages and fried food, at least once a week, whilst only about 10% visited fast food restaurants with the same frequency. Moreover, our results showed that selected family factors might additionally increase this differentiation. Therefore, educational programs should take into account existing diversity in target groups and select high priority topics for total population but also for subgroups.

Breastfeeding is recommended by the World Health Organization (WHO) and the American Academy of Pediatrics (AAP), as the optimal way of feeding infants, especially exclusive breastfeeding, during the first six months of life and complementary breastfeeding until twelve months of age or longer [27,28]. Actual, documented barriers of breastfeeding in Poland include, a lack of knowledge about lactation among health care practitioners taking care of mothers and children, commonness of supplementation with artificial formulas, and a low availability of professional lactation support system after discharge from the hospital [29]. In our examined group, most mothers did not breastfeed as long as it is recommended. Almost half of the children were not breastfed at all or for no longer than three months. Our results were similar to those obtained in Polish nationwide epidemiological studies on childhood eating habit, conducted in 1997 [30] and in 2013 [29]. Studies conducted in different regions of Poland also reported similar results to our study [31,32]. In Europe, Poland ranks among the top ten countries, with a high percentage of women, who start breastfeeding after birth. The situation is worse when it comes to the total length of breastfeeding. In Poland, the median feeding length is 4.8 months, while in Norway it is equal to 12 months, and in Italy, Finland, and Germany, it is about 7 months [33]. The prevalence of breastfeeding at 12 months in most high-income countries is lower than 20%, but it varies significantly between the United Kingdom (<1%), the United States (27%), Norway (35%) [34], and Poland (11.9%) [33].

A significant influence of the number of children in the family and the mother’s education level on the duration of breastfeeding, was observed in our research. Research among mothers from East Poland (*N* = 262) showed that breastfeeding was assessed as “not very difficult” only by women who delivered at least two children [35]. In other studies, there was a statistically significant association between knowledge about WHO recommendations for the shortest period of exclusive breastfeeding and the number of children in the family. Respondents with two children were more likely to consider the appropriate time of breastfeeding to be “six months” than women with one, three, or more children; significant differences were observed. The same research showed that much more often the correct “six months” response was provided by women with higher education, in comparison to respondents with vocational or secondary education [36]. Relationship between better knowledge concerning breastfeeding and higher education level was also confirmed by other studies [37,38,39]. Our findings and other cited works suggest that a higher education level and having more experience in being a mother might promote a longer breastfeeding.

Nutritional recommendations aimed at preventing the NCDs include, maintaining an appropriate energy balance, limiting fat intake to 30% energy, saturated fatty acids to 10% energy, dietary cholesterol below 300 mg a day, and avoiding the intake of trans-unsaturated fatty acids from confectionery, fried fat, chocolate by-products, crisps, and fast food. In practice, children should limit the consumption of snacks, especially the sweet ones, as well as fast food and sugary drinks [40]. 

Some parents use sweets as a reward for good behavior or school performance. Using sweets as a form of reward teaches children bad eating behaviors for the future. If children receive sweets as a reward, the child will like them and also need them in adult life, and there’s a high probability that as adults, they will also reward themselves in the same way [41]. Only one other research in Poland concerning using sweets as a reward was conducted among children (*N* = 44; ages 8–13). In this study, 52.3% of children received chocolate as a reward, while 29.5% received ice-cream [42], which was similar to our results declared for all types of sweets. In our work, such a reward was more popular among parents with more children, which in our opinion could be associated with sweet uniqueness and a less availability resulting from a limited budget per child.

Poland is one of the few countries in Europe that failed to reduce the incidence of caries in children, despite WHO recommendations. Currently, it is estimated that the statistical twelve-year-old child in Poland has 3.5 teeth affected by caries and 40% of eighteen-year-olds have deficiencies in permanent teeth, due to dental caries complications [43]. Intensity of caries in Poland is five times higher than that in the United States and in the Western European countries, while frequency of caries is two–three times higher, respectively [18]. Despite the widespread prevalence of caries in Poland, this problem is often underestimated and a proper oral hygiene begins only in older children, when changes are often extensive and irreversible, requires dental procedures, and often require a root canal treatment, resulting in dead teeth and premature teeth loss [44]. Analyzing results based on mothers’ declarations, we concluded that the examined mothers probably had a low awareness of the presence of dental caries in their children and might have downplayed the problem. In the cited Ministry of Health document it was stated that the prevalence of dental caries increased with children’ age, while in our study the opposite relation was found. It is likely that the examined mothers monitored the younger children’s teeth more frequently or it could be an effect of the adult teeth eruption time, in the examined group. Higher prevalence of caries was reported in our study for children from villages, which is in line with the national data for twelve year-old children [18].

In our research, the majority of children consumed sweet beverages once a day, but many children consumed them even more often. This suggests an extensive sugar consumption. Similar results were reported by nationwide research among 6383 pupils aged 11–15 years. At least once a day sugary drinks were consumed by 25.4% children [45]. Even higher consumption was reported in other Polish studies among children from South Poland (*N =* 350; ages 10–12). Once a day sweet beverages were consumed by 37% of pupils [46]. However, this number is much lower than that in England (70%) [47], and that in the United States, where sweet beverages contribute 39% of all added sugars consumed by adolescents [48]. However, our results were worse in comparison to Germany, where such beverages are consumed by 25.2% of boys and 19.8% girls, respectively [49]. In our research, no influence of sex was observed on sweet beverage consumption. This is contrary to other studies, including the Health Behaviour in School-aged Children (HBSC) study from 2009–2010 which reported a higher sweet beverage consumption among boys [15,45,46,50,51]. We observed significant influence of age, place of residence, mother’s education level, and monthly net income level. According to other authors, and similar to our findings, younger children in Poland consumed less sweet beverages than the older ones [15]. In our examined population, children from villages consumed sweet beverages more frequently than those from the city. The same results were reported by other authors [45,46]. In our work, the higher the education level of the mother, the lower the consumption of sweet beverages observed. The opposite was reported by Suliga; in her work, mothers’ higher education was connected with higher consumption of sweet beverages [52]. This author’s research was conducted more than ten years ago and concerned only breakfast so it could be the reason for the different conclusion. Literature review of forty-four works attempted to provide sweet beverage consumption determinants. According to authors’ observations, low income, alike low education level of parents, promotes a higher consumption of such products [53].

Almost all mothers surveyed in our study admitted that their children consumed unhealthy snacks. This was in line with results of other studies from different regions of Poland that have also reported a high frequency of consumption of these product groups. In central Poland (*N =* 1100; ages 11–13), sweets were consumed a few times a week, by 74% of children, and salty snacks were consumed by 36% of them [54]. In Western Poland (*N* = 1263; ages 13–16) sweets were consumed, every day, by 38.2% pupils [55]. Even worse results were reported for Southern Poland (*N* = 350; ages 10–12), where at least 60% of the 350 children studied, consumed sweets each day [46], and Eastern Poland *(N =* 1829; ages 10–15), where 63% of pupils consumed them, at least once a day [56]. In Germany, chocolate and other sweets were eaten daily by 36% of children in a similar age range [49], while in the United States, sweet snacks accounted for up to 9% of the typical children’s daily caloric intake [57]. Salty snacks are generally consumed more rarely but results from East Poland *(N =* 220; ages 7–16) showed that 3.6% of children ate them every day, while 49.1% consumed them at least once a week [58]. 

In the examined population we observed a relation between the child’s sex and frequency of consumption of fried food. Boys consumed such products more frequently than girls. The same boys, according to our published previous findings, were significantly more often overweight than girls [25]. Extensive national research among 14,355 children aged 9–14 years, from 50 states of the United States, suggested that children who consumed greater quantities of fried food were heavier, had greater total energy intake, and had a poorer diet quality. In addition, increasing consumption of fried food over time, according to the authors, might lead to an excess weight gain [22]. 

Eating out-of-home food is usually associated with higher energy intake, compared to the consumption of food at home. In addition, research shows that more energy is consumed with meals eaten at fast food restaurants than at traditional restaurants [59]. The mothers examined in our research did not declare frequent food consumption (along with their children) at fast food restaurants. Quite similar results were reported by other studies. In Eastern Poland (*N* = 110; ages 7–13), according to the parents’ declarations, 7.3% of children consumed fast food, at least once a week, 19.1% consumed two to three times a month, 59.1% consumed very rarely, and 13.6% did not eat such food at all [58]. Additionally, parents from North Poland *(N =* 100; ages 5–10) declared that 26% of them did not eat with their children, at fast food restaurants, 36% of them did so less often than once a month, while, 20%, 10%, and 7% consumed fast food once a month, once a week, and more often than once a week, respectively [60]. Additionally, similar results were reported by the German Health Interview and Examination Survey for Children and Adolescents. According to the authors, three-quarters of children ate fast food once a month or less [49]. Similar frequency of consumption was also reported for Southern European countries [61]. Much frequent consumption was observed in the United States, where 30.3% children consumed fast food daily [62]. It should be strongly emphasized that the consumption of fast food, with a frequency higher than once a week, usually entails a low-quality diet and a BMI value closely related to the amount of fast food products consumed [63].

In our study, there was a significant influence of the place of residence, the mother’s educational level, monthly income per family, and the number of children in a family, on fast food consumption. Differentiated popularity of fast food consumption, in relation to the place of residence, has also been observed by other authors, who examined 282 pupils, aged 14, from Central Poland. Similar to our study, fast food was more popular in the urban areas where it was consumed by 60–80% of pupils, while in the village if the young consumed meals outside the home it was food from the school canteen [64]. Nevertheless, a national study among 13,150 students from the United States stated that fast food availability was not associated with weekly frequency of fast food consumption in non-urban, low- or high-density urban areas [65]. Similar to our research, a higher frequency of consumption of fast food among children of mothers with a lower education level was also found amongst two hundred pre-school children from Northern Poland. This study presented similar findings concerning family income. The higher the income, the higher the observed consumption of fast food. among children [66]. It is likely that, in Poland, children from the richest families are taken to fast food restaurants as such places are still perceived as prestigious.

A lack of statistically significant associations between the selected nutritional aspects and children’s BMI could be attributed to various individual susceptibility to overweight/obesity development. This might be connected mainly to genetic/epigenetic factors affecting the energy balance, total amount, and a variety of food consumed, adipogenic activity, energy expenditure, and an even circadian rhythmicity of metabolism [67,68,69,70,71,72]. Moreover, the methodology of nutritional assessment in our study should be taken into consideration. More quantitative approach, like an overall dietary intake assessment, could reveal distinct relations between unhealthy nutrition and the danger of being overweight.

Some limitations of this study are discussed. First, this study is a self-reported one and might contain several potential sources of partiality, especially the participant’s selective memory and social desirability bias. Second, as this was a cross-sectional research, there was no possibility to indicate any causality of the examined factors. Finally, it should also be noted that results could be site-specific, limited to regional studies.

## 5. Conclusions

Benefits resulting from breastfeeding for mother and the child should be more widely communicated. Special support should be given to mothers experiencing their first pregnancy, to whom breastfeeding is something completely new. Such actions might result in a larger number of women choosing to exclusively breastfeed or extend its duration.

The educational actions among, both, children and parents are necessary to promote healthy eating habits and reduce the consumption of snacks, sweet beverages, and fried food. During meetings with parents, educational materials promoting the principles of a balanced diet in the prevention of caries and NCDs, should be provided, including guidelines on preparing meals without frying or reducing the frequency of serving sweet beverages and snacks. 

Our results suggest that efforts should be continued to ensure that children, especially in rural areas, have free access to drinking water at schools, during class times. In the school canteens, fried food and sweet beverages should be avoided.

Extensive activities to reduce the occurrence of dental caries are necessary as many mothers are likely not aware of the prevalence of this problem. A good solution could be a dental care plan for both children and adolescents, including obligatory dental examinations and tooth-brushing exercises.
